# Differential Effects of 2-Hydroxypropyl-Cyclodextrins on Lipid Accumulation in *Npc1*-Null Cells

**DOI:** 10.3390/ijms21030898

**Published:** 2020-01-30

**Authors:** Sanzana Hoque, Yuki Kondo, Nodoka Sakata, Yusei Yamada, Madoka Fukaura, Taishi Higashi, Keiichi Motoyama, Hidetoshi Arima, Katsumi Higaki, Akio Hayashi, Takaki Komiya, Yoichi Ishitsuka, Tetsumi Irie

**Affiliations:** 1 Department of Clinical Chemistry and Informatics, Graduate School of Pharmaceutical Sciences, Kumamoto University, 5-1 Oe-honmachi, Chuo-ku, Kumamoto 862-0973, Japan; shusmita_huq@yahoo.com (S.H.); ykondo@kumamoto-u.ac.jp (Y.K.); nodokkan@gmail.com (N.S.); 162y3101@st.kumamoto-u.ac.jp (Y.Y.); 171y2002@st.kumamoto-u.ac.jp (M.F.); 2 Program for Leading Graduate Schools “HIGO (Health life science: Interdisciplinary and Glocal Oriented) Program”, Kumamoto University, 5-1 Oe-honmachi, Chuo-ku, Kumamoto 862-0973, Japan; 3 Priority Organization for Innovation and Excellence, Kumamoto University, 5-1 Oe-honmachi, Chuo-ku, Kumamoto 862-0973, Japan; higashit@kumamoto-u.ac.jp; 4 Department of Physical Pharmaceutics, Graduate School of Pharmaceutical Sciences, Kumamoto University, 5-1 Oe-honmachi, Chuo-ku, Kumamoto 862-0973, Japan; motoyama@gpo.kumamoto-u.ac.jp; 5 Laboratory of Evidence-based Pharmacotherapy, Daiichi University of Pharmacy, 22-1 Tamagawa-machi, Minami-ku, Fukuoka 815-8511, Japan; h-arima@daiichi-cps.ac.jp; 6 Research Initiative Center, Organization for Research Initiative and Promotion, Tottori University, 86 Nishi-cho, Yonago 683-8503, Japan; kh4060@med.tottori-u.ac.jp; 7 Discovery Research Laboratories, Ono Pharmaceutical Co., Ltd., 3-1-1 Sakurai Shimamoto-cho, Mishima-gun, Osaka 618-8585, Japan; a.hayashi@ono.co.jp (A.H.); t.komiya@ono.co.jp (T.K.)

**Keywords:** Niemann–Pick disease type C, cyclodextrin, 2-hydroxypropyl-β-cyclodextrin, 2-hydroxypropyl-γ-cyclodextrin, miglustat, cholesterol, lysosome, sphingolipid, sphingomyelin

## Abstract

Niemann–Pick disease type C (NPC) is an autosomal recessive disorder characterized by abnormal accumulation of free cholesterol and sphingolipids in lysosomes. The iminosugar miglustat, which inhibits hexosylceramide synthesis, is used for NPC treatment, and 2-hydroxypropyl-β-cyclodextrin (HP-β-CD), a cyclic oligosaccharide derivative, is being developed to treat NPC. Moreover, therapeutic potential of 2-hydroxypropyl-γ-cyclodextrin (HP-γ-CD) was shown in NPC models, although its mechanism of action remains unclear. Here, we investigated the effects of HP-β-CD, HP-γ-CD, and their homolog 2-hydroxypropyl-α-cyclodextrin (HP-α-CD) on lipid accumulation in *Npc1*-null Chinese hamster ovary (CHO) cells compared with those of miglustat. HP-β-CD and HP-γ-CD, unlike HP-α-CD, reduced intracellular free cholesterol levels and normalized the lysosome changes in *Npc1*-null cells but not in wild-type CHO cells. In contrast, miglustat did not normalize intracellular free cholesterol accumulation or lysosome changes in *Npc1*-null cells. However, miglustat decreased the levels of hexosylceramide and tended to increase those of sphingomyelins in line with its action as a glucosylceramide synthase inhibitor in both *Npc1*-null and wild-type CHO cells. Interestingly, HP-β-CD and HP-γ-CD, unlike HP-α-CD, reduced sphingomyelins in *Npc1*-null, but not wild-type, cells. In conclusion, HP-β-CD and HP-γ-CD reduce the accumulation of sphingolipids, mainly sphingomyelins, and free cholesterol as well as lysosome changes in *Npc1*-null, but not in wild-type, CHO cells.

## 1. Introduction

Niemann–Pick disease type C (NPC) is a rare autosomal recessive disease resulting in a lysosomal lipid trafficking dysfunction caused by mutations in either of the two genes encoding lipid-transporting proteins, *NPC1* or *NPC2* (95% and 5% of patients, respectively) [1,2,3]. Soluble luminal protein NPC2 and transmembrane protein NPC1 play important roles in transporting cholesterol from late endosomes/lysosomes to other organelles such as the endoplasmic reticulum and plasma membrane [2,4,5]. Therefore, dysfunction of the NPC1/2 protein disrupts this transport system, leading to abnormal accumulation of cholesterol and sphingolipids in endolysosomal compartments. This results in manifestations such as neurological dysfunction and liver and lung failure [1,6].

Miglustat, N-butyl-deoxynojirimycin, is an approved drug for the treatment of NPC in Europe, Canada, and Japan, which inhibits the rate-determining enzyme for glucosylceramide synthesis hexosylceramide synthase. It reduces the intracellular levels of hexosylceramide as well as of GM2 and GM3 gangliosides. Miglustat has been shown to delay neurological symptoms and prolong lifespans [7,8,9] in NPC animal models, suppress neurological deficits, such as gait disorder, dysphagia, cataplexy, and dystonia, and improve ambulation and swallowing abilities in patients with NPC [10,11,12,13,14]. Recently, Colaco et al. [15] reported that miglustat appears to have a therapeutic potential against Tangier disease, a metabolic disease caused by a defect in ATP-binding cassette transporter 1, which can be misdiagnosed as NPC.

The compound 2-hydroxypropyl-β-cyclodextrin (HP-β-CD), a cyclic oligosaccharide containing seven glucose units, which forms inclusion complexes with hydrophobic compounds such as cholesterol, has been identified as an attractive drug candidate for NPC treatment [16,17,18,19,20,21]. HP-β-CD has been reported to show beneficial effects, such as life prolongation, lysosomal volume decrease, and improved intracellular cholesterol trafficking, in NPC animal models and cells [22,23,24]. Because HP-β-CD is used as a pharmaceutical additive to solubilize hydrophobic drugs in parenteral and liquid formulations [25], it has also been used to treat patients with NPC compassionately and is undergoing clinical trials in the US [26] and European Union [27].

We recently reported the therapeutic potential of 2-hydroxypropyl-γ-cyclodextrin (HP-γ-CD), which has eight glucose units and a possible better safety profile than HP-β-CD, in NPC patient-derived cells and NPC model mice [28]. Davidson et al. [29] also confirmed the therapeutic potential of HP-γ-CD by animal experiments, although they observed little interaction with cholesterol compared with HP-β-CD in an aqueous solution. Therefore, the mechanisms by which HP-γ-CD attenuates lipid abnormalities, including cholesterol accumulation, in NPC pathology remain unclear. Additionally, several studies have demonstrated the benefits of HP-β-CD and HP-γ-CD for cholesterol metabolism, but the effect of cyclodextrins on sphingolipid accumulation is poorly understood.

Therefore, we conducted the present study to examine the comparative effects of cyclodextrin derivatives and miglustat on abnormal lipid metabolism such as cholesterol and sphingolipid accumulation induced by NPC pathology. We compared the effects of three hydroxypropyl-cyclodextrins, i.e., HP-β-CD, HP-γ-CD, and 2-hydroxypropyl-α-cyclodextrin (HP-α-CD, which has six glucose units), and miglustat by measuring free cholesterol, sphingolipids—such as sphingomyelin and hexosylceramide—and LysoTracker^®^ fluorescence intensity—as a measure of lysosomal expansion following lipid accumulation—in *Npc1*-null Chinese hamster ovary (CHO) cells.

## 2. Results

### 2.1. Effects of Cyclodextrins and Miglustat on LysoTracker^®^ Fluorescence Intensity and Intracellular Cholesterol Level in Npc1-Null CHO Cells

**Figure 1:**
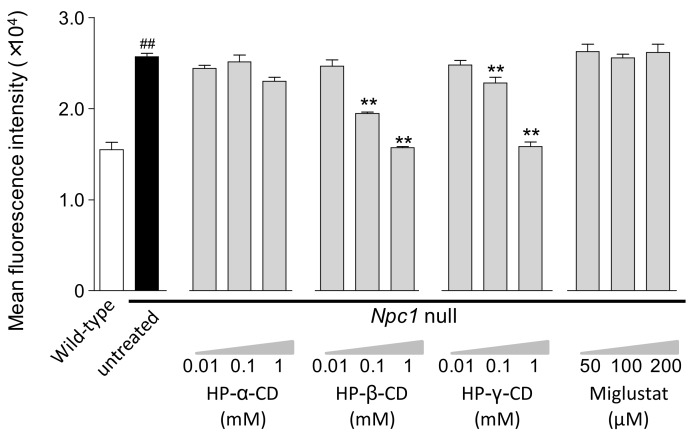
Concentration-dependent effects of cyclodextrins (HP-α-CD, HP-β-CD, and HP-γ-CD) and miglustat on impaired lysosomal compartments in *Npc1*-null CHO cells. The mean fluorescence intensity of LysoTracker^®^ was determined by flow cytometry at 24 h after exposure to cyclodextrins or miglustat. Each bar represents the mean ± S.E.M; (*n* = 3), ^##^ *p* < 0.01 compared with the wild-type (WT) group, ** *p* < 0.01 compared with the untreated *Npc1*-null group.

Previously, we demonstrated that HP-β-CD and HP-γ-CD attenuate the increase in LysoTracker^®^ fluorescence intensity and the abnormal cholesterol trafficking in *Npc1*-null CHO cells and neural and hepatocyte progenitors derived from induced pluripotent stem (iPS) cell lines from NPC patients [23,28]. Here, we first confirmed the effects of three cyclodextrin derivatives and miglustat on impaired lysosomal compartments in *Npc1*-null CHO cells by measuring lysosomal changes using LysoTracker^®^. The fluorescence intensity of LysoTracker^®^ was significantly higher in *Npc1*-null CHO cells than wild-type (WT) CHO cells. Treatment with HP-β-CD and HP-γ-CD reduced the fluorescence intensity in a dose-dependent manner, with a maximum effect observed at a concentration of 1 mM, at which the fluorescence intensity was almost the same as that in WT CHO cells. However, HP-α-CD and miglustat showed no effect at any concentration (Figure 1). We also found little effects of the drugs on the fluorescence intensity of LysoTracker^®^ in WT CHO cells (data not shown).

**Figure 2:**
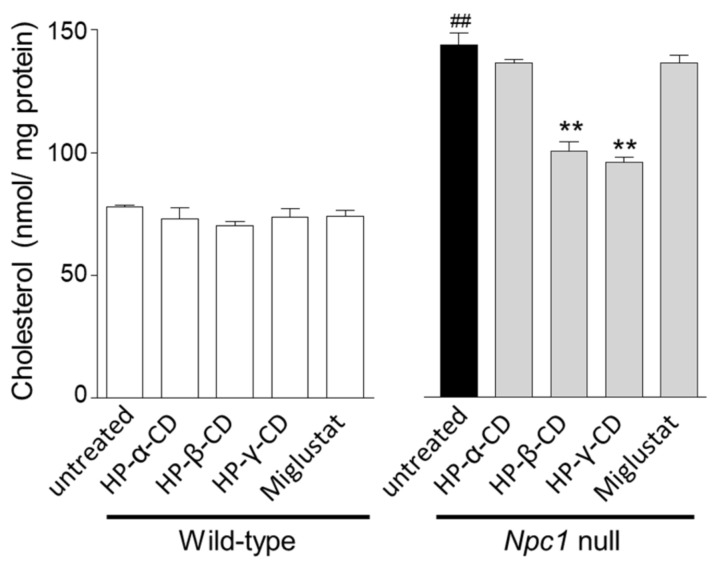
Effects of cyclodextrins (HP-α-CD, HP-β-CD, and HP-γ-CD) and miglustat on cellular free cholesterol levels in WT and *Npc1*-null CHO cells. Cellular free cholesterol levels were measured 24 h after exposure to cyclodextrins (1 mM) or miglustat (100 μM). Each bar represents the mean ± S.E.M. (*n* = 3); ^##^ *p* < 0.01 compared with the WT group, ** *p* < 0.01 compared with the untreated *Npc1*-null group.

Next, we evaluated the effects of cyclodextrins and miglustat on free cholesterol accumulation in *Npc1*-null CHO cells. *Npc1*-null CHO cells showed higher cholesterol levels compared with WT CHO cells. HP-α-CD and miglustat did not improve abnormal cholesterol trafficking, but HP-β-CD and HP-γ-CD (1 mM) significantly decreased cholesterol levels in *Npc1*-null CHO cells (Figure 2, right). The cholesterol levels of WT CHO cells were indistinguishable in all treatment groups (Figure 2, left).

### 2.2. Effects of Cyclodextrins and Miglustat on Intracellular Sphingolipid Levels

**Figure 3:**
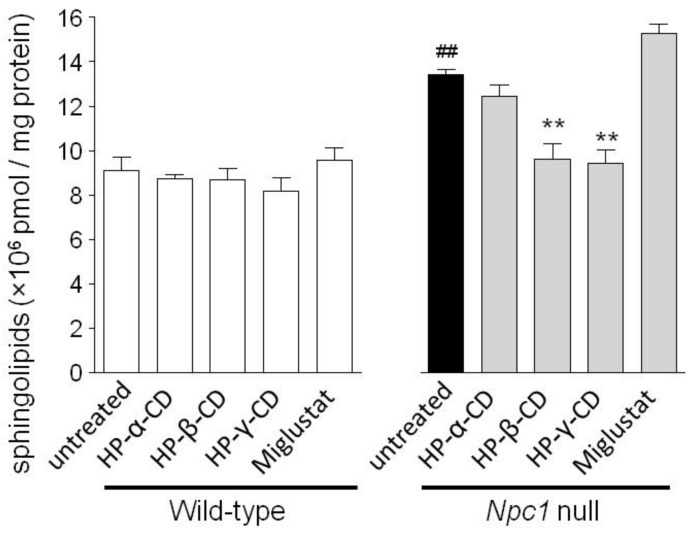
Effects of cyclodextrins (HP-α-CD, HP-β-CD, and HP-γ-CD) and miglustat on total sphingolipid levels in WT and *Npc1*-null CHO cells. Total sphingolipid levels were measured 24 h after exposure to cyclodextrins (1 mM) or miglustat (100 μM). Each bar represents the mean ± S.E.M. (*n* = 3); ^##^ *p* < 0.01 compared with the WT group, ** *p* < 0.01 compared with the untreated *Npc1*-null group.

We evaluated changes in sphingolipid levels by the treatments in both WT and *Npc1*-null CHO cells. As shown in Figure 3, total sphingolipid levels (sphingomyelin, hexosylceramide, and ceramide) in *Npc1*-null CHO cells were significantly higher than in WT CHO cells. HP-β-CD and HP-γ-CD (1 mM) reduced the total intracellular sphingolipid level in *Npc1*-null CHO cells, but both cyclodextrins showed little effect in WT CHO cells. No significant effects were observed in Npc1-null or WT CHO cells treated with HP-α-CD and miglustat. 

Next, we analyzed the changes in the levels of each sphingolipid following treatment with cyclodextrins or miglustat. Sphingolipids contain fatty acid chains in the ceramide skeleton. Therefore, various molecular species of sphingolipids are present depending on the number of carbon atoms and unsaturated double bonds. Supplemental Table S1 lists the sphingolipids measured in this study. We measured various molecular species of ceramides, sphingomyelins, and hexosylceramides in both WT and *Npc1*-null CHO cells using tandem mass spectrometry and then analyzed their levels using volcano plots. In *Npc1*-null CHO cells, we observed larger amounts of sphingomyelins compared with WT CHO cells. Although some hexosylceramides were also elevated in *Npc1*-null CHO cells compared with WT cells, ceramide levels were almost identical in *Npc1*-null and WT CHO cells (Supplemental Figure S1).

**Figure 4:**
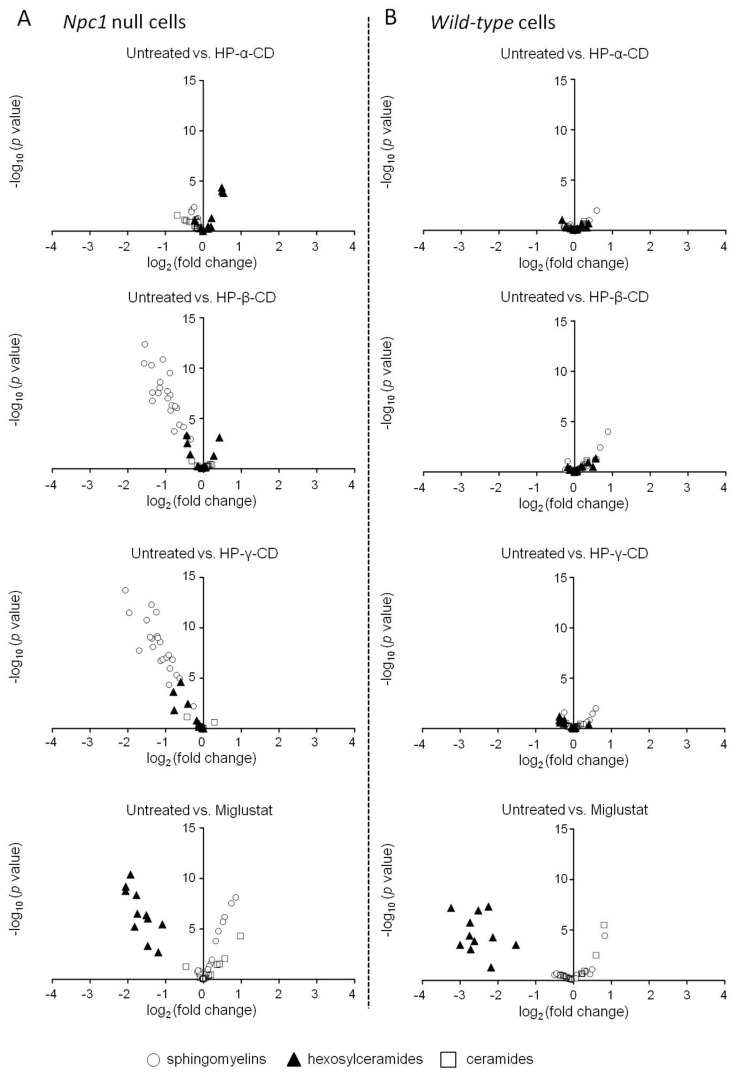
Effects of cyclodextrins (HP-α-CD, HP-β-CD, and HP-γ-CD) and miglustat on sphingolipid levels in WT (**B**) and *Npc1*-null (**A**) CHO cells. Sphingolipid levels were measured 24 h after treatment with 1 mM of each cyclodextrin and 100 µM of miglustat in *Npc1*-null and WT CHO cells. The x-axis shows the logarithm to base 2 of fold changes in each sphingolipid level induced by the treatments. The y-axis shows the negative logarithm to base 10 of *t*-test *p*-values. Open circle: sphingomyelins, closed triangle: hexosylceramides, open square: ceramides.

We also compared the effects of cyclodextrins and miglustat on intracellular sphingolipids in *Npc1*-null and WT CHO cells. Treatment with HP-β-CD and HP-γ-CD significantly reduced sphingomyelin levels in *Npc1*-null CHO cells, whereas HP-α-CD showed no such effects (Figure 4A). None of the cyclodextrins affected the levels of any sphingolipid in WT CHO cells (Figure 4B). However, miglustat significantly reduced hexosylceramide levels and tended to increase sphingomyelin levels in both *Npc1*-null and WT CHO cells (Figure 4, bottom). In addition, we performed a comparison between untreated WT cells and reagent-treated *Npc1*-null CHO cells in terms of sphingolipid changes (Supplemental Figure S2). Although HP-β-CD- and HP-γ-CD-treated *Npc1*-null CHO cells showed little statistical significance, significant differences in the sphingolipid changes were observed in HP-α-CD- and miglustat-treated *Npc1*-null CHO cells compared with untreated WT CHO cells. Detailed data are shown in Supplemental Material File S1.

## 3. Discussion

In this study, we evaluated the comparative effects of 2-hydroxypropyl-cyclodextrins and miglustat on free cholesterol and sphingolipid contents in *Npc1*-null and WT CHO cells. We found that HP-β-CD and HP-γ-CD, but not HP-α-CD, significantly attenuated sphingolipid accumulation, particularly sphingomyelins, as well as free cholesterol accumulation and lysosomal expansion in *Npc1*-null CHO cells. Although miglustat reduced hexosylceramide levels, it increased sphingomyelin levels and had no effect on free cholesterol or LysoTracker^®^ fluorescence intensity in *Npc1*-null CHO cells. These data indicate that HP-β-CD and HP-γ-CD exert similar effects on lipid composition in *Npc1*-null CHO cells and that their mode of action differs from that of miglustat.

The attenuating potentials of HP-β-CD in terms of abnormal lipid accumulation and lysosome volume in NPC model cells have been reported in an earlier study [23]. Moreover, we previously observed comparable effects of HP-γ-CD and HP-β-CD against cholesterol accumulation and autophagy dysfunction in NPC patient-derived cells [28]. In this study, we confirmed the reducing effects of HP-β-CD and HP-γ-CD, unlike HP-α-CD, on free cholesterol and LysoTracker^®^ fluorescence intensity in *Npc1*-null CHO cells. Our data indicate that both cyclodextrins have attenuating potentials against NPC in our experimental system. Under these conditions, we observed that HP-β-CD and HP-γ-CD significantly decreased sphingomyelin levels in *Npc1*-null CHO cells but had little effect on the levels of hexosylceramides and ceramides. 

Cholesterol can coexist with sphingomyelins in the cellular membrane [30]; they appear to play some physiological roles together, while sphingomyelins contribute to cholesterol accumulation in NPC disease [31]. This suggests a possible relationship between free cholesterol and sphingomyelin reduction by HP-β-CD. Additionally, Long et al. [32] found that HP-β-CD ameliorates sphingomyelin accumulation and lysosomal expansion in cells from patients with Niemann–Pick disease type A, another lysosomal storage disease with cellular sphingomyelin accumulation caused by acid shingomyelinase inactivity. These facts support our findings of HP-β-CD and HP-γ-CD attenuation of sphingomyelin accumulation in *Npc1*-null CHO cells. Although the role of sphingomyelin accumulation in the development of NPC manifestations, such as hepatosplenomegaly, is unknown, our results suggest that the reductions of both free cholesterol and sphingomyelin accumulation by HP-β-CD and HP-γ-CD are involved in resolving these manifestations.

When considering the reducing effects of cyclodextrins on sphingolipids, a physicochemical evaluation of their molecular interaction may be valuable. In a previous study, we demonstrated that HP-β-CD and HP-γ-CD form inclusion complexes with sphingomyelin and enhance its solubility in a phosphate buffer system, which corresponds to the present findings. However, HP-α-CD has a greater potential for sphingomyelin solubilization than HP-β-CD and HP-γ-CD [33], despite the fact that it does not reduce sphingomyelin accumulation in *Npc1*-null CHO cells. These data indicate that the reduction of sphingomyelins by HP-β-CD and HP-γ-CD is caused by an indirect effect rather than by a direct interaction with sphingolipids. Further study is warranted to clarify the mechanism.

Miglustat, an approved drug for the treatment of NPC in Europe, Canada, and Japan, inhibits hexosylceramide synthase and reduces the levels of intracellular hexosylceramides as well as of GM2 and GM3 gangliosides [34,35,36,37]. The reducing effects on hexosylceramides and gangliosides appear to be critical for the neuroprotective effects of miglustat [38]. In this study, 100 µM miglustat significantly decreased intracellular hexosylceramide levels in *Npc1*-null and WT CHO cells. Therefore, we considered that this dose was sufficient in our experimental system. Indeed, treatment with 50–200 µM miglustat had little effect on free cholesterol accumulation and lysosome abnormalities in *Npc1*-null CHO cells, suggesting that miglustat cannot attenuate abnormal cholesterol trafficking or impaired vesicle transport caused by NPC. This is consistent with previous findings using iPS cells from NPC patients [39]. In addition, the lowering effects on hexosylceramides in WT CHO cells suggest unfavorable actions of miglustat, such as adverse reactions. Schlegel et al. [40] reported that treatment with miglustat impairs spatial learning in WT mice compared with a sham group. Further basic and clinical studies to clarify this issue are warranted.

The volcano plot analysis of intracellular sphingolipid contents in *Npc1*-null and WT CHO cells indicated that HP-β-CD and HP-γ-CD attenuated sphingolipid accumulation in *Npc1*-null cells, with little effect on WT cells. However, HP-α-CD showed no effects, and miglustat significantly reduced hexosylceramide levels in both *Npc1*-null and WT CHO cells. These findings are supported by the results from the comparison between untreated WT cells and reagent-treated *Npc1*-null CHO cells. The results indicated that HP-β-CD and HP-γ-CD, unlike HP-α-CD, had attenuating effects on the sphingolipid abnormality related to *Npc1* gene deficiency. Interestingly, the lack of effect of HP-β-CD or HP-γ-CD in WT CHO cells also suggested pathophysiological selectivity of HP-β-CD and HP-γ-CD and low toxicity of HP-β-CD and HP-γ-CD related to sphingolipid metabolism. 

In this study, we demonstrated the effects of cyclodextrins and miglustat on cholesterol and sphingolipid accumulation in *Npc1*-null CHO cells. However, this study has the following limitations. First, the results were derived from only one representative cell model, *Npc1*-null CHO cells. Considering the typical manifestations of NPC, such as neuronal injury and hepatitis, data from neuronal or hepatic NPC cell models would be needed. The *Npc1*-null CHO cell system was developed by our colleagues Higaki et al. [41], who suggested that these cells would be a useful tool to study the regulation of cellular cholesterol homeostasis and pathogenesis of NPC. This cell system has been used in numerous NPC studies [42,43,44] and proved to be useful in NPC research such as candidate drug screening. However, little was known about changes in the sphingolipid contents of *Npc1*-null CHO cells. Therefore, our study revealed the usefulness of this cell system for the evaluation of sphingolipid and cholesterol changes in NPC pathophysiology. Of course, to evaluate the neuroprotective or hepatoprotective actions of cyclodextrins and miglustat, further studies using other NPC model cells are needed, such as neural or hepatic progenitors derived from iPS cell lines of NPC patients [28]. Second, it is still unclear which kind of lysosomal change is measured by determining LysoTracker^®^ fluorescence intensity in NPC model cells. In our study, the LysoTracker^®^ fluorescence intensity was increased significantly in *Npc1*-null CHO cells compared with WT CHO cells, and treatment with HP-β-CD and HP-γ-CD attenuated the changes with high reproducibility. Previous reports have also used LysoTracker^®^ fluorescence intensity as a marker of lysosome volume expansion [45,46]. However, there is little information about the correlation between fluorescence intensity and lysosomal size in the previous and current studies. Further study is needed to clearly reveal the meaning of changes in fluorescence intensity. In addition, a comparative evaluation of LysoTracker^®^ fluorescence intensity and immunofluorescence staining of lysosomal marker proteins, such as lysosomal-associated membrane protein (LAMP) 1 and LAMP2, in our *Npc1*-null CHO cells is needed. Third, the dose-dependent effects of HP-β-CD and HP-γ-CD on sphingolipid contents is still unclear. In previous reports, we confirmed that HP-β-CD shows the same dose-dependent prevention as measured by the determination of both LysoTracker^®^ fluorescence intensity and dysfunction of cholesterol trafficking in *Npc1-*null CHO cells, and the maximum effects were observed at around 1 mM HP-β-CD. In this study, we reconfirmed the maximum effects for 1 mM HP-β-CD in the LysoTracker^®^ experiments and adopted this concentration for sphingolipid measurement. However, it is unclear whether 1 mM HP-β-CD and HP-γ-CD exert maximum effects on sphingolipid metabolism in our experimental system. To confirm the appropriate concentration and dose-dependency of cyclodextrins, further study is needed. 

## 4. Materials and Methods

### 4.1. Reagents

HP-α-CD (MW: 1164.53, D.S.: 3.3), HP-β-CD (MW: 1402.38, D.S.: 4.61), and HP-γ-CD (MW: 1563.92, D.S: 4.6) were provided by Nihon Shokuhin Kako Co., Ltd. (Tokyo, Japan). Miglustat (N-butyldeoxynojirimycin HCl) (MW: 255.74) was purchased from Carbosynth Ltd. (Compton, UK). Determiner L FC was obtained from Kyowa Medex Co., Ltd. (Tokyo, Japan). Dulbecco’s modified Eagle’s medium (DMEM), F-12, penicillin, streptomycin, and trypsin were obtained from Gibco (Life Technologies Japan, Tokyo, Japan). Lysotracker^®^ Green DND-26 and HyClone™ fetal bovine serum (FBS) were purchased from Thermo Fisher Scientific Inc. (Waltham, MA, USA) and BIowest (Nuaillé, France), respectively. The number of cells was counted by a Luna automatic counter (Logos Biosystems, Anyang, South Korea). Deionized and biopure distilled water was used in this study; all other reagents were of commercially available reagent grade.

### 4.2. Cell Culture

Cellular experiments were performed as described in our previous studies [23,47]. WT and *Npc1-*null CHO cells [41] were grown in culture medium consisting of a 1:1 mixture of DMEM/F12 supplemented with 10% FBS, 100 IU/mL penicillin, and 100 mg/mL streptomycin and maintained in an incubator at 37 °C with 5% CO_2_. For all assays, cells were cultured for at least 24 h in 96-well plates, 6-well plates, or 10 cm dishes, depending on the experiment. Cyclodextrins and miglustat were dissolved in the same culture medium (DMEM/F12 supplemented with FBS and antibiotics) and administered to the cells in the following experiments.

#### 4.2.1. Measurement of the LysoTracker^®^ Fluorescence Intensity

We evaluated the effects of cyclodextrins and miglustat on lysosomal changes as NPC manifestations using LysoTracker^®^ Green DND-26. WT and *Npc1*-null CHO cells were seeded at 1 x 10^5^ cells /well in 12-well plates and incubated at 37 °C for 24 h to allow cell adhesion. The cells were then treated with or without cyclodextrins and miglustat for 24 h. We confirmed that 8–24 h of treatment with HP-β-CD exerted sufficient and significant attenuating effects against NPC in our preliminary results (data not shown). The concentration ranges of cyclodextrins and miglustat were based on our previous studies [23,28]. The cells were washed with phosphate-buffered saline and stained with 50 nM LysoTracker^®^ dissolved in DMEM/F12 (1:1) with 10% FBS and antibiotics for 15 min at 37 °C. Then, the cells were collected by trypsinization, and LysoTracker^®^ fluorescence was measured by a flow cytometer (BD Biosciences Accuri™ C6, Becton Dickinson Biosciences, Franklin Lakes, NJ, USA) using a 488 nm laser. Finally, using a 530 ± 30 nm filter, fluorescence was detected in the FL1 channel. Data from 10,000 cells were collected and represented as the mean fluorescence intensity using BD Accuri C6 software (Becton Dickinson Biosciences). We performed three independent experiments (N = 3) for each group and show the mean value and standard error of the mean (SEM) of the three values. 

#### 4.2.2. Measurement of Intracellular Cholesterol Levels

Intracellular cholesterol levels were measured according to our previous reports [23,47]. WT and *Npc1*-null CHO cells were seeded in 6-well plates at 2 ×10^5^ cells/well. Both cell lines were incubated in medium with or without cyclodextrins and miglustat for 24 h. They were then lysed, and the protein concentration was measured using a Pierce BCA Protein Assay kit (Thermo Fischer Scientific Inc.). Cholesterol was extracted from the lysate using a mixture of chloroform, 2-propanol, and NP-40 (7:11:0.1). Samples were centrifuged at 15,000 × *g* for 10 min at 4 °C, and then the bottom chloroform layers were collected. After evaporation of the chloroform layers, the residues were dissolved in a mixture of 2-propanol, polyoxylene alkyl ether, and polyoxylene lauryl ether (87:10:3). The cholesterol content in the samples was measured by a Determiner L FC (Kyowa Hakko Kirin Co., Ltd., Tokyo, Japan).

#### 4.2.3. Measurement of the intracellular sphingolipid level by High-Performance Liquid Chromatography–Tandem Mass Spectrometry (LC–MS/MS)

WT and *Npc1*-null CHO cells were seeded at 1 × 10^5^ cells/10 cm dish and precultured for 24 h at 37 °C. They were then incubated in medium containing 100 μM miglustat and 1 mM cyclodextrin derivatives for 24 h at 37 °C. After trypsinization, the cells were collected and centrifuged at 4700× *g* for 10 min. Then, a suspension of 1 × 10^6^ cells/mL was prepared, and 1 mL was centrifuged at 4700× *g* for 10 min. The cell pellet was dissolved in 50 μL RIPA buffer (FUJIFILM Wako Pure Chemical Corporation, Osaka, Japan). Afterwards, the BCA protein assay was performed on the lysate, the protein concentration was adjusted to 1 mg/mL, and the solution was used for lipid determination. Each sphingolipid was measured by LC–MS/MS with the Acquity UPLC system coupled with a 4000QTRAP triple quadrupole mass spectrometer (AB Sciex Pte. Ltd., Tokyo, Japan) equipped with an electrospray ionization source as described previously [48]. Briefly, the Acquity UPLC BEH-C18 reverse-phase column (1.7 μm particle size, 1.0 mm i.d. × 100 mm) was used for sample separation at 50 °C. Samples were injected as 4 μL volumes at a flow rate of 0.080 mL/min. Mobile phases A (0.5 mM CH_3_COONH_4_ and 0.1 mM H_3_PO_4_) and B (acetonitrile/methanol 4:1) were applied for gradient elution as follows: 0–1 min: 27% B, 1–2 min: increased linearly to 66% B, 2–10 min: 85% B, 10–16 min 100% B, maintained at 100% B for 13 min, and finally returned to the initial phase. The Scheduled MRM™ Pro Algorithm was used for lipid monitoring. Internal standards were purchased from Avanti Polar Lipids (Alabaster, AL, USA). The total sphingolipid content was estimated by summing all measured sphingolipids, as shown in Table S1.

#### 4.2.4. Statistical Analysis

Multiple comparisons were made to examine the data of lysosomal changes measured by LysoTracker^®^ fluorescence, intracellular free cholesterol, and total sphingolipids ( Figure 1 Figure 2 Figure 3, respectively). We used GraphPad Prism ver. 5.01 (GraphPad Software, San Diego, CA, USA) for the calculations. When equal variances of the data were identified by Bartlett’s test (*p* < 0.05), one-way analysis of variance was used to test for statistical differences. If significant differences (*p* < 0.05) were identified, the results were analyzed further by Tukey’s multiple range test for significant differences among the values. Comparison of each sphingolipid level between two groups was performed by volcano plot analysis. The statistical significance was examined using the unpaired Student’s *t*-test by JMP Pro 14.4.4 (SAS Institute Inc., Cary, NC, USA) was used.

## 5. Conclusions

In conclusion, we demonstrated that miglustat did not reduce total sphingolipid levels but rather increased sphingomyelin and ceramide and reduced hexosylceramide levels in both *Npc1*-null and WT CHO cells. These results suggest that miglustat changes the sphingolipid composition but cannot reduce the total amount of lipids in *Npc1*-null CHO cells. However, HP-β-CD and HP-γ-CD, unlike HP-α-CD, attenuated the accumulation of sphingolipids, particularly sphingomyelins, and free cholesterol as well as lysosome expansion in *Npc1*-null CHO cells, whereas little effect was seen in WT CHO cells. These results provide a rational basis for the dosage regimens required to optimize the therapeutic efficacy of combined drug use in NPC patients. 

## Supplementary Materials

The following are available online at https://www.mdpi.com/1422-0067/21/3/898/s1.

## Abbreviations

NPC	Niemann–Pick disease type C
CD	Cyclodextrin
HP-β-CD	2-Hydroxypropyl-β-cyclodextrin
HP-γ-CD	2-Hydroxypropyl-γ-cyclodextrin
HP-α-CD	2-Hydroxypropyl-α-cyclodextrin
CHO	Chinese hamster ovary
WT	Wild-type
S.E.M.	Standard error of the mean
DMEM	Dulbecco’s modified Eagle’s medium
FBS	Fetal bovine serum
BCA	Bicinchoninic acid
LC/MS/MS	High-performance liquid chromatography–tandem mass spectrometry
iPS	Induced pluripotent stem
LAMP	Lysosomal-associated membrane protein
