# Supplementary material for: Differential Effects of 2-Hydroxypropyl-Cyclodextrins on Lipid Accumulation in Npc1-Null Cells
Source: Int J Mol Sci. 2020 Jan 30;21(3):898. doi: 10.3390/ijms21030898 (PMC7038050; doi:10.3390/ijms21030898)
Supplement: Supplementary file 1 [file ijms-21-00898-s001.pdf]

Table S1. List of sphingolipids detected and quantified in this study.

Cer(18:1/16:0)	SM(18:1/16:0)	SM(18:1/23:0)	HexCer(18:1/16:0)
Cer(18:1/18:0)	SM(18:1/18:0)	SM(18:1/23:1)	HexCer(18:1/18:0)
Cer(18:1/20:0)	SM(18:1/18:1)	SM(18:1/24:0)	HexCer(18:1/20:0)
Cer(18:1/22:0)	SM(18:1/19:0)	SM(18:1/24:1)	HexCer(18:1/22:0)
Cer(18:1/22:1)	SM(18:1/20:0)	SM(18:1/24:2)	HexCer(18:1/22:1)
Cer(18:1/23:0)	SM(18:1/20:1)	SM(18:1/26:1)	HexCer(18:1/24:0)
Cer(18:1/24:0)	SM(18:1/21:0)	SM(18:1/26:2)	HexCer(18:1/24:1)
Cer(18:1/24:1)	SM(18:1/21:1)	SM(18:0/22:0)	HexCer(18:1/24:2)
Cer(18:1/24:2)	SM(18:1/22:0)	SM(18:0/22:1)	HexCer(18:1/26:0)
Cer(18:1/25:0)	SM(18:1/22:1)	SM(18:0/24:1)	HexCer(18:1/26:1)
Cer(18:0/16:0)	SM(18:1/22:2)		HexCer(18:0/24:0)

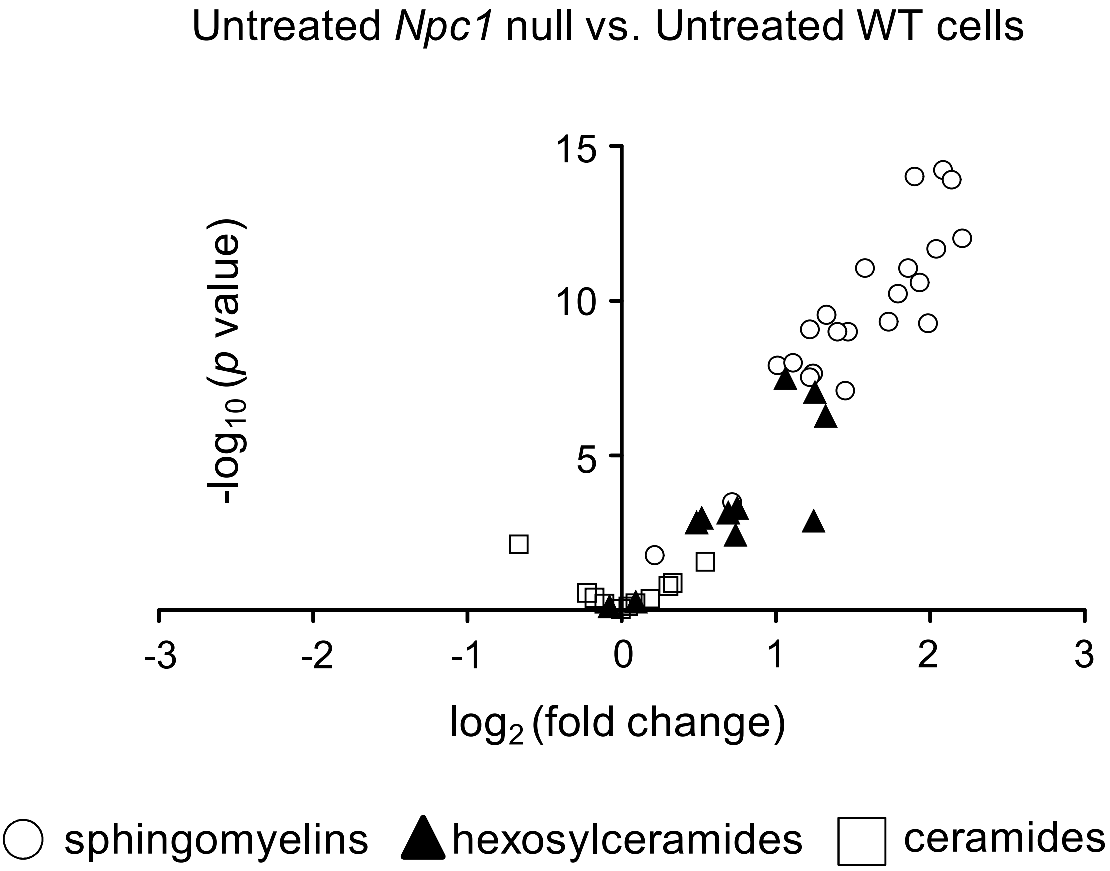

Figure S1. Comparison of cellular sphingolipid content between *Npc1*-null and WT CHO cells. The x-axis shows the logarithm to base 2 of fold changes in each sphingolipid level of *Npc1*-null CHO cells compared with WT CHO cellsThe y-axis shows the negative logarithm to base 10 of t-test *p*-values. Open circle: sphingomyelins, closed triangle: hexosylceramides, open square: ceramides.

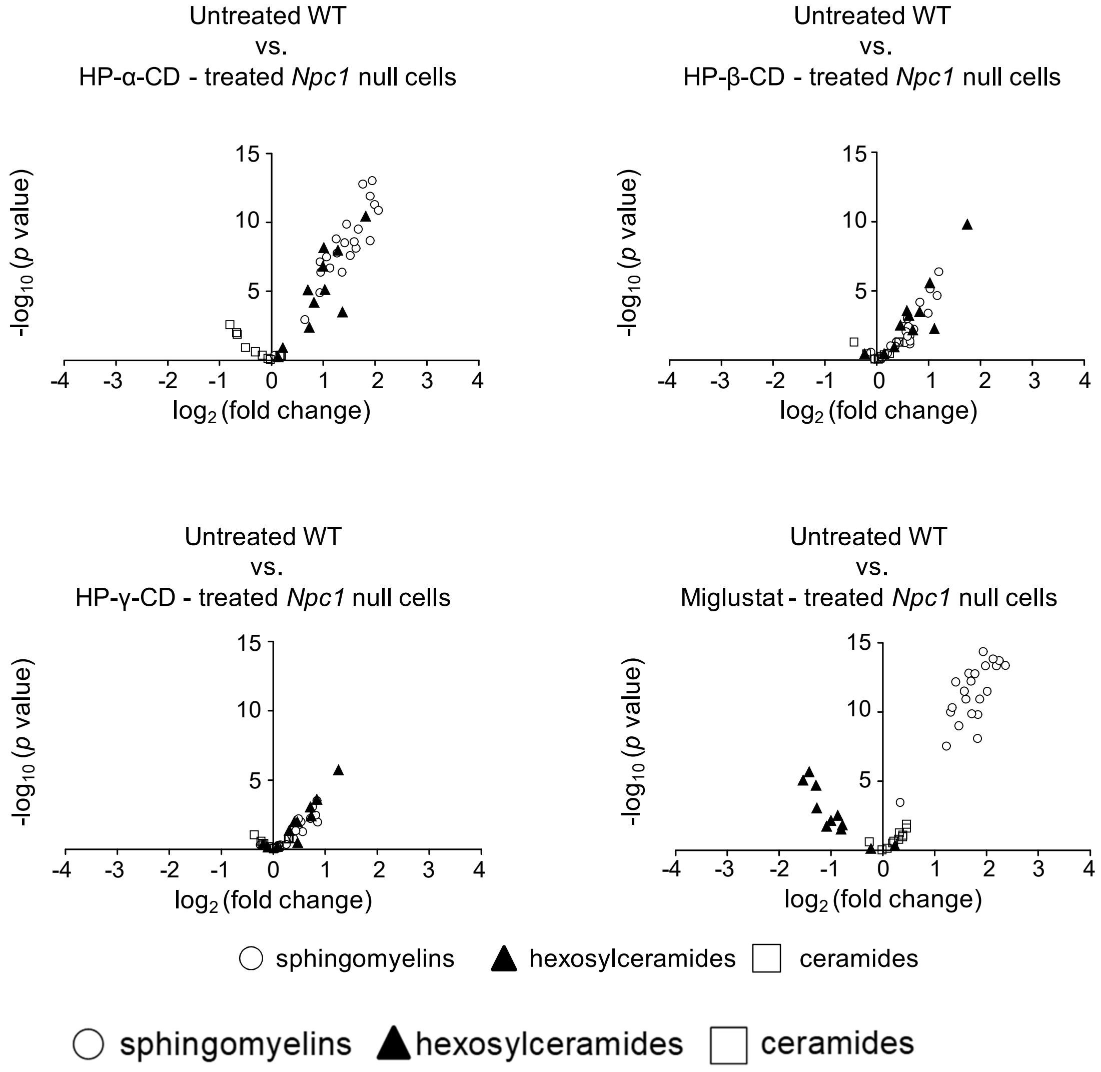

Figure S2. Comparison of cellular sphingolipid contents between WT CHO cells and treated *Npc1*-null cells. The x-axis shows the logarithm to base 2 of fold changes in each sphingolipid level of treated *Npc1*-null CHO cells compared with WT CHO cells. The y-axis shows the negative logarithm to base 10 of t-test *p*-values. Open circle: sphingomyelins, closed triangle: hexosylceramides, open square: ceramides.
